# Thermal and Sliding Wear Properties of Wood Waste-Filled Poly(Lactic Acid) Biocomposites

**DOI:** 10.3390/polym14112230

**Published:** 2022-05-30

**Authors:** Tej Singh, Amar Patnaik, Lalit Ranakoti, Gábor Dogossy, László Lendvai

**Affiliations:** 1 Savaria Institute of Technology, Faculty of Informatics, ELTE Eötvös Loránd University, 9700 Szombathely, Hungary; sht@inf.elte.hu; 2 Department of Mechanical Engineering, Malaviya National Institute of Technology, Jaipur 302017, Rajasthan, India; apatnaik.mech@mnit.ac.in; 3 Mechanical Engineering Department, Graphic Era (Deemed to be University), Dehradun 248002, Uttarakhand, India; lalit_9000@yahoo.com; 4 Department of Materials Science and Engineering, Széchenyi István University, 9026 Győr, Hungary; dogossy@sze.hu

**Keywords:** poly(lactic acid), wood waste, biocomposite, sliding wear, microscopy

## Abstract

In our study, the effects of wood waste content (0, 2.5, 5, 7.5, and 10 wt.%) on thermal and dry sliding wear properties of poly(lactic acid) (PLA) biocomposites were investigated. The wear of developed composites was examined under dry contact conditions at different operating parameters, such as sliding velocity (1 m/s, 2 m/s, and 3 m/s) and normal load (10 N, 20 N, and 30 N) at a fixed sliding distance of 2000 m. Thermogravimetric analysis demonstrated that the inclusion of wood waste decreased the thermal stability of PLA biocomposites. The experimental results indicate that wear of biocomposites increased with a rise in load and sliding velocity. There was a 26–38% reduction in wear compared with pure PLA when 2.5 wt.% wood waste was added to composites. The Taguchi method with L_25_ orthogonal array was used to analyze the sliding wear behavior of the developed biocomposites. The results indicate that the wood waste content with 46.82% contribution emerged as the most crucial parameter affecting the wear of PLA biocomposites. The worn surfaces of the biocomposites were examined by scanning electron microscopy to study possible wear mechanisms and correlate them with the obtained wear results.

## 1. Introduction

Strict government regulations and increased environmental constraints on the burning and open-air dumping of agricultural, municipal, and industrial wastes have encouraged material scientists to develop innovative products such as biocomposites [1,2,3]. Several studies have highlighted that biocomposites hold the potential to be used in various applications, including automotive, infrastructure, aerospace, construction, consumer, and industrial fields [1,2,3,4,5]. According to reports, the forest industry was assumed to play a significant part in the economy of any country and generated a revenue of 270 billion USD worldwide in 2018 [6]. Solid wood waste is the main by-product of the forest industry, with more than 14 million tons of wood waste being generated every year, which is a significant problem when it comes to disposal [7,8]. Additionally, it was reported that more than 70 million tons of solid wood waste were generated annually during the manufacturing of wood products [9]. Furthermore, the annual production of synthetic plastics exceeds 320 million tons, which is unfortunately accumulated in productive agricultural parcels as landfills or incinerated in open-air [10]. Reducing wood waste and synthetic plastic can be achieved by replacing synthetic plastics with bioplastics to develop biocomposites. The global biocomposite market size was 16.46 billion USD in 2016, and it is estimated to reach 36.76 billion USD by 2022 with a compound annual growth rate of 14.44% [11].

Numerous scientists have examined how to improve the various properties of biocomposites, including thermal, thermomechanical, and mechanical features, by utilizing distinctive waste materials [12,13]. The potential of sugarcane bagasse and maize hull agro-wastes in thermoplastic starch-based biocomposites was studied by Dogossy and Czigany [14]. The influence of industrial wastes, namely copper slag and drill cuttings, on thermal, physical, mechanical, and antibacterial properties of poly(ε-caprolactone)-based biocomposites was investigated by Hejna et al. [15]. Kim et al. [16] studied the mechanical properties and biodegradability of rice husk and wood waste-filled poly(butylene succinate)-based biocomposites. Panaitescu et al. [17] evaluated the thermal and mechanical properties of wood waste-reinforced poly(3-hydroxybutyrate) biocomposites. Dhakal et al. [18] examined the influence of date palm fiber waste biomass on the mechanical properties of polycaprolactone-based biocomposites.

In recent years, there has been a remarkable interest in poly(lactic acid) (PLA) for many reasons, such as increasing environmental awareness, reducing product prices by capacity growth, and taking advantage of good processability [19,20]. PLA is a bio-based polymer, originating from the fermentation of corn, sugar beet, potatoes, and other agriculture-based substances. The major advantages of PLA are its biodegradability under certain temperature/pressure conditions and its non-toxic nature. It has good stiffness and strength compared with synthetic polymers, and it can be altered and adjusted for a wide range of applications, including packaging, textile, and biomedical purposes [21,22,23,24,25,26]. However, PLA has its drawbacks as well, including rapid physical aging, poor impact resistance, relatively high price, and low thermal stability. These drawbacks associated with PLA can be overcome by adopting, blending, copolymerization, or adding some materials such as filler/reinforcement [27,28,29,30].

Khan et al. [31] studied hemp hurd’s impact on the mechanical properties of PLA-based biocomposites. They concluded that the evaluated tensile and flexural strength of the manufactured biocomposites decreased, whereas crystallinity and the tensile and flexural modulus improved by increasing hemp hurd content. They also pointed out that with glycidyl methacrylate grafting, the biocomposites with ≥20 wt.% hemp hurd loading demonstrated mechanical properties nearly equal to bare PLA. Orue et al. [32] explored the potential of alkali-treated walnut shells waste as filler for the PLA matrix. They incorporated alkali-treated walnut shell powder (10, 20, and 30 wt.%) into the matrix for biocomposite fabrication. A relative improvement of 50% in tensile strength was reported for treated walnut shell waste-filled biocomposites compared with the untreated counterpart. However, the tensile modulus values of treated walnut shell waste-filled biocomposites remained almost similar to unfilled PLA, and they maintained a consistent behavior with increased filler content. Boubekeur et al. [33] investigated the influence of 1–3 mm sized wood waste (a mixture of eucalyptus and Aleppo pine wood) particles on the mechanical properties of PLA-based biocomposites. The authors concluded that Young’s modulus and crystallinity of the manufactured composites increased, while stress, impact strength, and elongation at break decreased with increasing wood waste percentage. Bajpai et al. [34] investigated the wear performance of natural fiber-reinforced PLA composites. Three categories of natural fibers (nettle, sisal, and *Grewia optiva*) were applied, and laminated composites were fabricated as per a hot compression process. The experimental results showed that incorporating natural fiber mats into the PLA matrix as a reinforcement remarkably enhanced the wear resistance of the neat polymer. There was a 10–44% decrease in friction coefficient and about 70% decrease in the specific wear rate of manufactured composites compared with neat PLA. Kanakannavar et al. [35] studied the effect of natural fiber 3D braided woven fabric as reinforcement in PLA composites for tribological performance. The research concluded that the fabric reinforcement decreased the specific wear rate of PLA, and about a 95% decrease was detected in the samples containing 35 wt.% reinforcement.

Although the literature is rich in research of the impacts of wood waste on the physical, mechanical, thermal, and thermo-mechanical properties of PLA-based biocomposites, the sliding wear behavior of wood waste-filled PLA-based biocomposites has not been studied so far. Moreover, the inclusion of some natural fibers and sustainable biocarbon was reported to enhance the wear resistance of PLA-based biocomposites [34,35,36]. Therefore, our research studied the production of PLA biocomposites using North Indian rosewood waste and investigating their thermal and dry sliding wear properties.

## 2. Experimental Details

### 2.1. Materials and Biocomposite Fabrication

**Figure 1:**
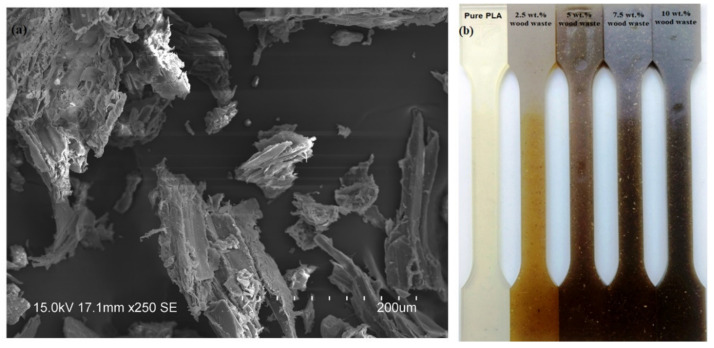
(**a**) SEM micrograph of North Indian rosewood waste, (**b**) fabricated biocomposites.

PLA (Nature Works, USA, Ingeo 2003D grade) with a melt flow index of 6 g/10 min, a density of 1.24 g/cm^3^, and a melting temperature of 170 °C was used in this research. North Indian rosewood waste (60 mesh) was procured from the Krishna Timber Store in Dadhol, Himachal Pradesh, India. Before use, the wood waste was treated with 2% sodium hydroxide solution for 12 h at room temperature. After that, the treated wood waste was washed with distilled water and dried in an oven for 4 h. The SEM (scanning electron microscope) micrograph of the North Indian rosewood waste particles is presented in Figure 1a. Before biocomposite manufacturing, both the wood waste and PLA were dried for 6 h in a DEGA-2500 dehumidifier at 80 °C. The melt compounding/mixing of the PLA biocomposites containing 0, 2.5, 5, 7.5, and 10 wt.% of North Indian rosewood waste was performed using an LTE 20–44 twin-screw extruder (Labtech Engineering, Samut Prakarn Thailand; L/D ratio of 44; screw diameter of 20 mm) with a screw speed of 30 rpm and a temperature profile of 155–185 °C. After melt compounding, the composites were cooled and granulated. Subsequently, the granulated samples were injection molded into dumbbell-shaped samples (Figure 1b) using an Arburg Allrounder Advance 420C (Loßburg, Germany) injection molding machine with a nozzle temperature of 195 °C. The following parameters were used for the injection molding process: injection rate of 40 cm^3^/s, holding pressure at 75-65-25 MPa for 15 s, cooling time of 30 s, and mold temperature at 30 °C [37].

### 2.2. Thermogravimetric Analysis

The thermogravimetric tests were performed on a Shimadzu TGA-50 model scientific instrument, while the evaluation was performed using TA-60WS software. The powdered sample (~10 mg) of biocomposites was placed in a platinum pan. The thermal stability was recorded at a heating rate of 10 °C min^−1^ in a nitrogen atmosphere from 30 °C to 500 °C.

### 2.3. Sliding Wear Study

The sliding wear behavior of the produced biocomposites was investigated utilizing an ASTM G-99-compliant pin on disk machine (Model: TR-411, DUCOM, India). The schematic of the pin on disk machine and its detailed working principles were discussed elsewhere [38]. A 20 mm × 5 mm × 5 mm specimen was machined from the manufactured composites, and it was held stationary within the fixture, which was normal to the disk. For load-speed sensitivity, a series of trials were carried out by varying the normal load (10 N, 20 N, and 30 N) and the sliding velocity (1 m/s, 2 m/s, and 3 m/s) on the pin on disk machine for a fixed sliding distance of 2000 m. The biocomposite sample weight was measured prior to and after the wear test by utilizing an electronic weight balance (Wensar Weighing Scales Ltd., India) with an accuracy of 0.0001 g. For each sample, the wear experiment was repeated three times, and the volumetric wear in cm^3^ was computed by using the following equation [37]:

(1) $$Volumetric\;wear\;=\frac{\varpi }{\rho }$$

where ϖ = sample weight loss (g) and ρ = sample density (g/cm^3^).

The density of the manufactured biocomposites was determined by using standard water displacement method, and it was found to be 1.24 g/cm^3^, 1.225 g/cm^3^, 1.211 g/cm^3^, 1.198 g/cm^3^, and 1.183 g/cm^3^ [39].

### 2.4. Experiment Design

In this study, the combination of control parameters for sliding wear minimization was determined using the Taguchi method. The Taguchi method is one of the most important statistical techniques used to demonstrate the influence of different control parameters with various levels. The sliding wear tests on the manufactured biocomposites were conducted under various working conditions, using four control parameters each with five levels: wood waste content (A: 0, 2.5, 5, 7.5, and 10 wt.%), normal load (B: 10, 20, 30, 40, and 50 N), sliding distance (C: 500, 100, 1500, 2000, and 2500 m) and sliding velocities (D: 0.6, 1.2, 1.8, 2.4, and 3 m/s) (as listed in Table 1).

In a full factorial design, nearly 625 (5^4^) trials would be required to contemplate the impact of four control parameters, each having five levels. In contrast, the Taguchi method decreases the number of trials by utilizing orthogonal arrays, resulting in a lower number of trials with noticeable precision. Therefore, the impact of four control parameters with five levels (as presented in Table 1) was studied using L_25_ orthogonal design as presented in Table 2. Further on, to assess the test results, the signal-to-noise (SN) ratio was also investigated. The Taguchi method has three categories of the SN ratio, namely lower-the-better, nominal-the-better, and higher-the-better. In this work, a ‘lower-the-better’ characteristic was utilized, since the intention was to minimize the wear by using the following equation [38].

(2) $$\mathrm{SN}\mathrm{ratio}=-10\log\left[\frac{1}{n}\left(\sum_{n}y^{2}\right)\right]$$

where *y* = volumetric wear and *n* = number of trials.

### 2.5. Contribution Ratio Analysis

After the SN ratio analysis, each control parameter was analyzed for their contribution ratio (ψ) towards the volumetric wear by using the following steps [40].

*Step I:* Calculation of the overall SN ratio mean. In this step the overall SN ratio mean (ℜ) was computed for the 25 trials using the following equation:

(3) $$ℜ=\frac{1}{25}\sum_{n=1}^{25}\left(SN\;ratio\right)$$

*Step II:* Level mean of the SN ratio. In this step, the level mean of the SN ratio ($\hbar_{i}$) was calculated for each control parameter using the following equation.

(4) $$\hbar_{i}=\frac{1}{5}\sum_{j=1}^{5}{\left(SN\;ratio\right)}_{ij}$$

where *j* is the level of the *i*th control parameter.

*Step III:* Sum of squares calculation. In this step, the sum of squares (ƛ) values were determined using the following equation:

(5) $$ƛ=\sum_{i}{\left(\hbar_{i}-ℜ\right)}^{2}$$

The *i*th control parameter can be determined using the following equation:

(6) $${ƛ}_{i}=\sum_{j=1}^{5}{\left(\hbar_{i}-ℜ\right)}^{2}$$

where *j* is the level of the *i*th control parameter.

*Step IV:* Contribution ratio calculation. In the final step, the contribution ratio (ψ) of the individual control parameter was calculated by using the following equation:

(7) $$\psi_{i}=\frac{{ƛ}_{i}}{ƛ}\times 100$$

### 2.6. Scanning Electron Microscopy

The worn surfaces of pure PLA and wood waste-filled PLA biocomposites were further examined for possible wear mechanisms using a Hitachi S-3400N scanning electron microscope (SEM; Hitachi Ltd., Tokyo, Japan). Prior to the SEM inspection, the samples were sputter-coated with a gold–palladium alloy in order to prevent charging.

## 3. Results and Discussion

### 3.1. Thermal Stability Analysis

**Figure 2:**
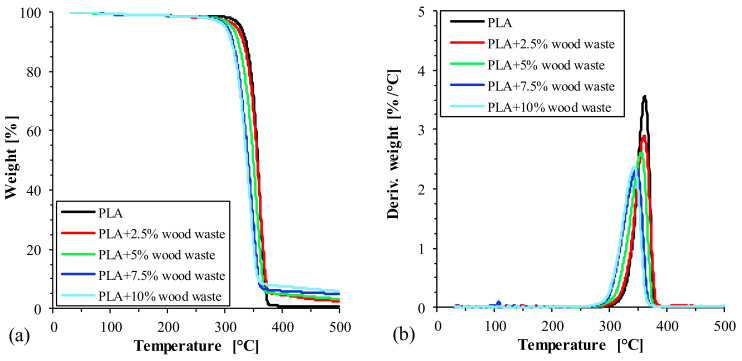
TGA (**a**) and DTG (**b**) curves for PLA biocomposites, respectively.

The temperature-dependent weight loss curves and the corresponding derivatives (DTG) for pure PLA and its wood waste-filled biocomposites are illustrated in Figure 2a,b, respectively. The thermal deterioration at temperatures ranging from 30 °C to 250 °C resulted in a minor weight loss of about 2 ± 0.5%. The elimination of moisture was the primary cause of the biocomposites’ weight loss at this point. A single-step decomposition process was observed both for the bare PLA and the wood waste-filled PLA biocomposites as well, in the range of (250–400 °C). The weight loss in this temperature range corresponded to the degradation of the PLA resin and the decomposition of hemicellulose, cellulose, and lignin that were present in the wood waste [41]. The temperature corresponding to the 5%, 25%, 50%, and 75% weight loss (i.e., T_5_, T_25_, T_50_, and T_75_) and the temperature of the maximum decomposition rate (T_peak_) are presented in Table 3. Based on the results, the thermal degradation of wood waste-filled PLA biocomposites occurred at a lower temperature than that of pure PLA. Biopolyesters such as PLA tend to degrade at elevated temperatures as a consequence of various depolymerization processes and thermal oxidation reactions [42]. The incorporation of the less thermally stable wood waste into the polymer matrix facilitated the thermal degradation of PLA, thereby leading to an earlier decomposition of the biocomposites during the heating.

Using 5% weight loss (T_5_) as the onset of the main degradation step, the temperature was 327 °C for the bare PLA but decreased to 320 °C when 2.5 wt.% wood waste was added. When the wood waste loading was increased even further (5, 7.5, and 10% wt.%), the composites’ onset degradation temperature decreased to 312 °C, 304 °C, and 300 °C, respectively. Similarly, with increased wood waste content, the temperatures for 25%, 50%, and 75% weight loss and the temperatures corresponding to the highest decomposition rate also decreased considerably. Previous studies reported similar trends with various natural fiber-filled PLA composites [42,43,44].

### 3.2. Influence of Normal Load and Sliding Velocity on Wear

**Figure 3:**
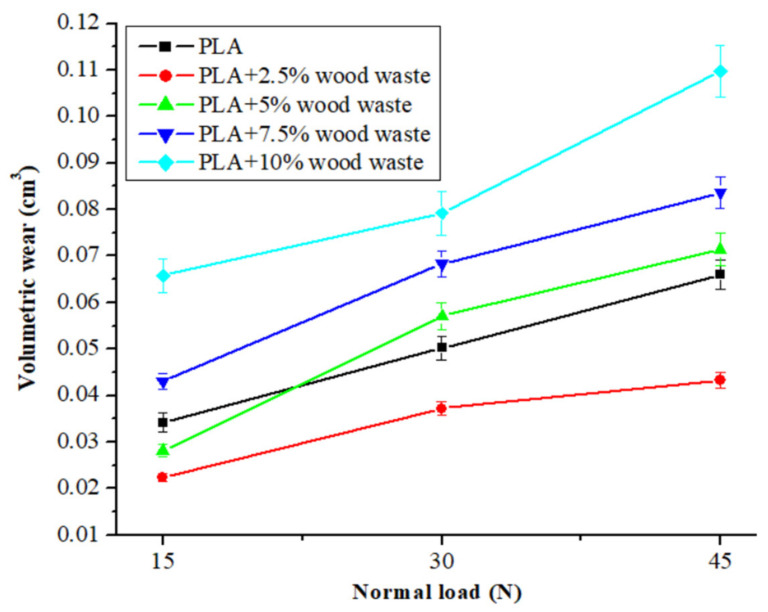
Volumetric wear of composite as a function of normal load.

Figure 3 shows the volumetric wear of composites as a function of normal load (10 N, 20 N, and 30 N) at a constant sliding velocity (1 m/s) and a 2000 m of fixed distance. Figure 3 shows that when the normal load increased, the volumetric wear of all composites increased dramatically. The volumetric wear fluctuated between 0.0342 cm^3^ and 0.0660 cm^3^ in pure PLA samples. Compared with pure PLA, the trend in volumetric wear for 2.5 wt.% wood waste-filled composite was modest, with an increased normal load. Adding 2.5 wt.% of wood waste reduced the volumetric wear of the PLA matrix by 26% to 34% under all loading situations. With the further addition of wood waste ≥ 5 wt.%, the wear of the composites increased, and it was the highest (0.0658–0.1097 cm^3^) when 10 wt.% wood waste was added to the composites. The possible mechanism for the increment in volumetric wear with increased wood waste content and normal load can be explained. The lower the wood waste particle concentration, the more that the structural homogeneities remained on the higher side due to ease in the dispersion of wood waste particles within the PLA matrix. The firm embedment of the wood waste particles helped to protect the matrix in the contact zone from heat and mechanical failure, resulting in minor wear.

**Figure 4:**
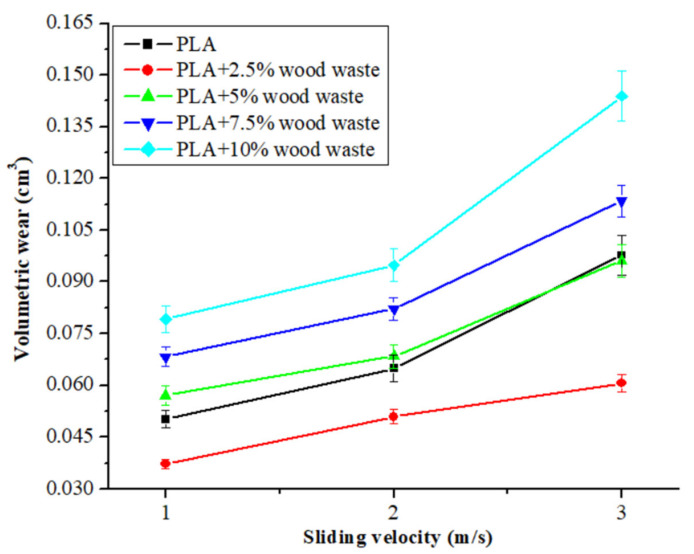
Volumetric wear of the composites as a function of sliding velocity.

After displaying a slight volumetric wear at 2.5 wt.% wood waste content, the volumetric wear was observed to rise when the wood waste loading was increased further. At higher concentrations, the possibilities of wood waste particles agglomerating expanded the composites and counter surface gap. As a result of the increasing distance, the adhesion between the sliding surfaces decreased, resulting in a more significant weight loss and volumetric wear. Additionally, with increased loading, the number of wood waste particles on the composite surface increased. As the normal load grew, more heat was generated during testing, and the interfacial contact temperature also increased. With this higher temperature, the bonding between the wood waste and the matrix weakened, and material removal became more accessible, increasing the wear. Similar results were reported by Bajpai et al. [34] for natural fiber-reinforced PLA composites and by Erdoğan et al. [45] for industrial waste-filled epoxy composites as well. Figure 4 shows the volumetric wear of composites as a function of sliding velocity (1 m/s, 2 m/s, and 3 m/s) at a constant normal load (30 N) and a 2000 m of fixed distance. The trend in volumetric wear for pure PLA was from 0.0502 cm^3^ to 0.0977 cm^3^; when wood waste was incorporated, the wear firstly decreased at 2.5 wt.% wood waste content and then increased with further wood waste loading. The volumetric wear remained at 0.0372–0.0606 cm^3^ for the 2.5 wt.% wood waste-filled composite, which was 26–38% lower than that of pure PLA. In comparison, the highest volumetric wear was registered for 10 wt.% wood waste-filled composites, which fluctuated between 0.0792–0.1439 cm^3^. The thermal softening of the PLA resin occurred as the sliding velocity rose due to increased heat production, resulting in increased wear with increased sliding velocity. The variation of volumetric wear with sliding velocity showed that the wear of the PLA composite increased when the sliding velocity rose higher. As the sliding velocity grew, the thermal softening of the PLA resin took place due to increased heat generation. The higher heat weakened the filler–resin bonding, and it became easier to detach the wood waste particles from the composite surface during sliding, which resulted in increased wear. Bajpai et al. [34] observed a similar mechanism for sliding wear in the case of natural fiber-reinforced PLA composites. For lower load-velocity sliding conditions, Megahed et al. [46] concluded that the generation of slight surface deformation resulted in lower wear. However, surface deformation increased at higher normal load and sliding velocity conditions, resulting in increased wear.

### 3.3. The Taguchi Analysis for Sliding Wear Performance

According to the literature, biocomposites can be used in various applications where wear is a critical issue. The wear performance of PLA biocomposites is significantly influenced by the type and amount of reinforcement and testing parameters [28,29,30]. Therefore, our investigation was designed to find the most significant control parameter and combination of parameters that yielded the slightest wear during sliding. The experiments were conducted as L_25_ orthogonal array design considering the impact of wood waste content, sliding distance, normal load, and sliding velocity on wear performance.

**Figure 5:**
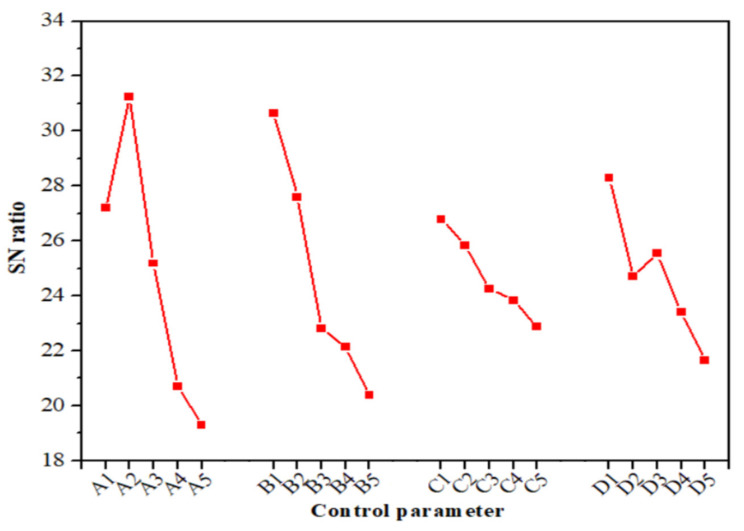
Main parameter effects for various SN ratio values of volumetric wear.

The Taguchi method suggests investigating the SN ratio by utilizing conceptual methodology that includes diagramming impacts and visually identifying the critical parameters. The results of the volumetric wear and their corresponding SN ratios are collected in Table 4. The investigation was conducted in Minitab 18. The volumetric wear obtained ranged from 0.0091 cm^3^ to 0.1727 cm^3^. The lowest and highest volumetric wear was obtained in test runs 6 and 25, respectively. Additionally, the influence of the selected four control parameters on the SN ratio of the volumetric wear is presented in Figure 5, while the SN ratio response is found in Table 5. As shown in Figure 5, there was a decrease in the volumetric wear of the composites upon increasing the amount of wood waste content from 0 to 2.5 wt.%; however, it started increasing above 2.5 wt.% wood waste content.

The minimum value of 0.0091 cm^3^ for volumetric wear was obtained for the 2.5 wt.% wood waste-filled composite. The situation changed when the wood waste content started increasing. The maximum value of 0.1727 cm^3^ was obtained for the biocomposite with 10 wt.% wood waste content. This significant behavior was potentially due to the agglomeration of the wood particles as a result of their poor interfacial bond with the PLA matrix. Due to the poor bonding, the wood waste particles were quickly drawn/peeled off from the PLA matrix during sliding, thus leading to the increased wear of the biocomposites. From the response displayed in Table 5, it can be assumed that among all the control parameters, wood waste content is an essential parameter, followed by normal load and sliding velocity, while sliding distance has a minimal impact on the volumetric wear of the tested biocomposite. Moreover, based on the results, it can be concluded that the combination of control parameters A_II_ (2.5 wt.% wood waste), B_I_ (10 N normal load), C_I_ (500 m sliding distance), and D_I_ (0.6 m/s sliding velocity) provided minimum volumetric wear. The result suggests that 2.5 wt.% wood waste-filled PLA biocomposite can be used for a low loading application of load and sliding velocity.

**Figure 6:**
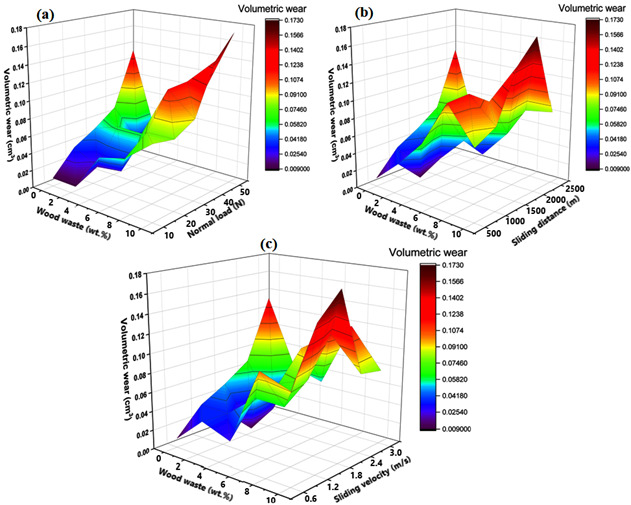
Contour plots of volumetric wear for wood waste content with respect to (**a**) normal load, (**b**) sliding distance, and (**c**) sliding velocity.

In addition, the influence of the most dominant control parameter (i.e., wood waste content) was analyzed on volumetric wear by drawing contour plots (Figure 6a–c) against (a) wood waste content and normal load, (b) wood waste content and sliding distance, and (c) wood waste content and sliding velocity. The contour plots demonstrate that the volumetric wear tended to increase when the wood waste content, normal load, sliding distance, and sliding velocity increased gradually. It was revealed that the lowest volumetric wear of 0.0091 cm^3^ was obtained at 2.5 wt.% wood waste content and the lower value (10 N) of the normal load. In contrast, the maximum volumetric wear of 0.1727 cm^3^ was obtained at 10 wt.% wood waste content and at a high level (50 N) of the normal load.

### 3.4. Contribution Ratio Results

**Figure 7:**
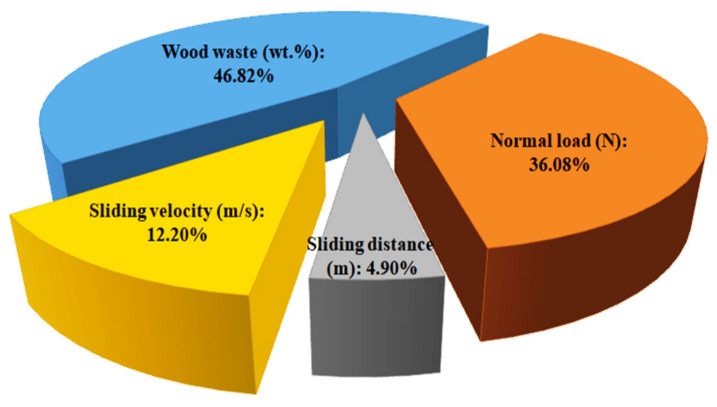
Contribution ratio of each control parameter to volumetric wear.

The contribution ratio of each parameter for volumetric wear is listed in Table 6 and presented in Figure 7. The overall SN ratio mean value (ℜ) for the 25 trials was determined using Equation (3) and found to be 24.72 dB. The level mean of SN ratio values for each control parameter was computed using Equation (4). The sum of squares (ƛ) value was determined by Equation (5), and for the individual control parameter the ${ƛ}_{i}$ value was determined by Equation (6). Thereafter, the contribution ratio (ψ) for each control parameter was computed using Equation (7). The results show that wood waste, normal load, sliding distance, and sliding velocity contributed to the volumetric wear by 46.82%, 36.08%, 4.90%, and 12.20%, respectively. The contribution results indicate that the wood waste content was the most significant control parameter affecting the volumetric wear of the biocomposites, followed by the normal load.

### 3.5. Worn Surface Morphology

**Figure 8:**
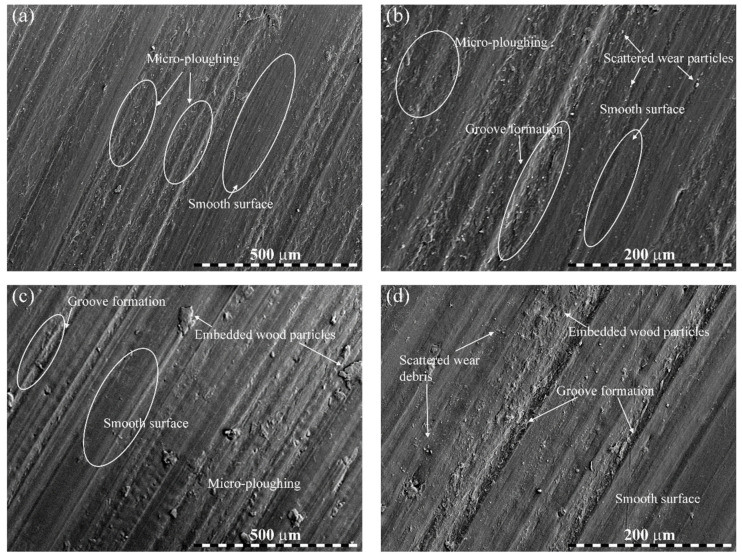
Worn micrographs of biocomposites: (**a**,**b**) bare PLA and (**c**,**d**) 2.5 wt.% wood waste-filled biocomposites at lower and higher magnification.

**Figure 9:**
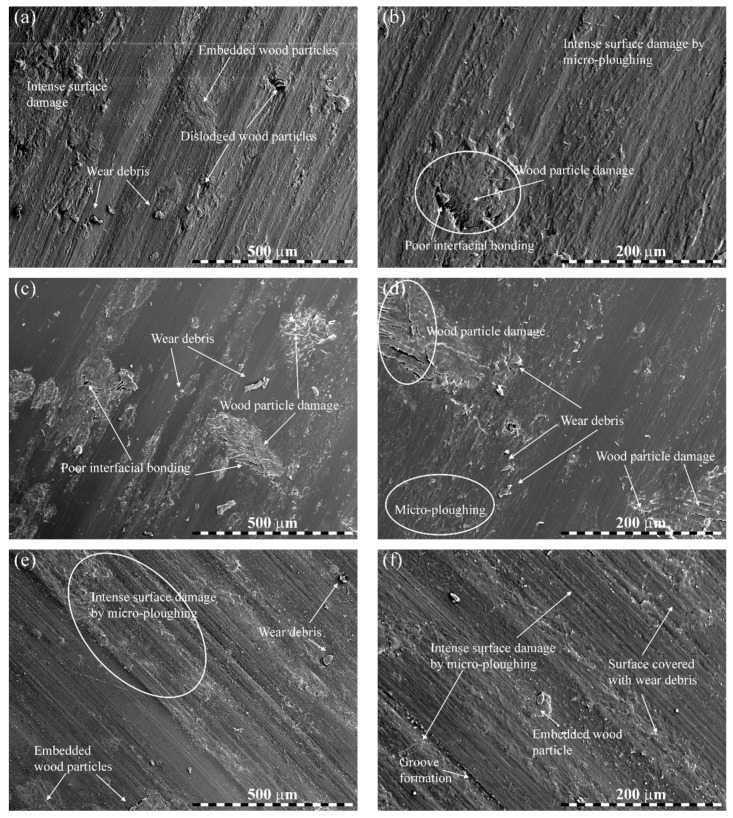
Worn micrographs of biocomposites: (**a**,**b**) 5 wt.%, (**c**,**d**) 7.5 wt.%, and (**e**,**f**) 10 wt.% wood waste-filled biocomposites at lower and higher magnification.

The results of SEM inspections are presented in Figure 8 and Figure 9. Figure 8a,b show the worn surfaces of bare PLA tested under 50 N load, 2500 m distance, and 3 m/s sliding velocity. In the image of lower magnification (Figure 8a), the worn surface was moderately rough, revealing possible micro-ploughing in the matrix. At a higher magnification (Figure 8b), the worn surface showed more scratches/damage to the matrix, resulting in increased material removal. As a consequence of sliding, the contact temperature was uncommonly expanded, which caused an accelerative rupture of the matrix, particularly in the interfacial zone. Accordingly, the surface damage strikingly expanded with grooves left by the matrix removal, resulting in a higher weight loss (Figure 8b). Figure 8c,d present the worn surfaces of 2.5 wt.% wood waste-filled biocomposite tested under 40 N load, 2500 m distance, and 0.6 m/s sliding velocity. In contrast with Figure 8a,b for bare PLA, the worn surfaces for 2.5 wt.% wood waste-added biocomposite was much smoother, and the matrix detachment was enormously restricted with the inclusion of wood waste particles. Even at lower magnification, the worn surface remained uniform with a lesser extent of micro-ploughing and groove formation, resulting in a slight wear of the biocomposite.

Figure 9a–f show the SEM images of the worn surfaces of 5 wt.% (under 50 N load, 1000 m distance, and at 2.4 m/s sliding velocity), 7.5 wt.% (under 40 N load, 1000 m distance, and at 3 m/s sliding velocity) and 10 wt.% (under 50 N load, 2000 m distance, and at 1.8 m/s sliding velocity) wood waste-filled PLA biocomposites. In comparison to Figure 8, the worn surfaces presented in Figure 9 were rougher with severe damage. Therefore, the worn surfaces were characterized by intense sub-surface damage due to sliding, while micro-ploughing was responsible for the heavy eradication of the surface material. The increased scattered wear particles and grooves formed by micro-ploughing contributed to the decreased wear resistance of these biocomposites. Moreover, the wood waste particles appeared to be seriously damaged, suggesting a poor filler-matrix interfacial bonding, which also resulted in elevated wear.

## 4. Conclusions

The thermal and sliding wear properties of Indian rosewood waste-filled PLA-based biocomposites were investigated. The following conclusions can be drawn:

1. The thermal stability of the PLA biocomposites increased with an increase in wood waste loading.
2. The wear of the biocomposites increased with an increase in load and sliding velocity. Compared with pure PLA, the wear in 2.5 wt.% wood waste-added biocomposites was almost 26–38% lower.
3. The Taguchi analysis demonstrated that the combination of control parameters A_II_ (wood waste of 2.5 wt.%), B_I_ (normal load of 10 N), C_I_ (sliding distance of 500 m), and D_I_ (sliding velocity of 0.6 m/s) offers the lowest volumetric wear for the manufactured biocomposites.
4. Wood waste content with 46.82% contribution was observed as the most dominant parameter for controlling the wear of the biocomposites, followed by the normal load, sliding velocity, and sliding distance with contributions of 36.08%, 12.20%, and 4.90%, respectively.
5. The worn surface study revealed that the micro-ploughing, grooves formation, and poor filler-matrix interfacial bonding were the possible cause of biocomposites wear.

## Figures and Tables

**Table 1:** Levels of the control parameters used in the experiment.

Control Parameters	Levels					Units
	I	II	III	IV	V	
A: Wood waste	0	2.5	5	7.5	10	wt.%
B: Normal load	10	20	30	40	50	N
C: Sliding distance	500	1000	1500	2000	2500	m
D: Sliding velocity	0.60	1.2	1.8	2.4	3	m/s

**Table 2:** Experimental design.

Test Run	Control Parameters				Test Run	Control Parameters			
	A	B	C	D		A	B	C	D
1	0.0	10	500	0.6	14	5.0	40	500	1.8
2	0.0	20	1000	1.2	15	5.0	50	1000	2.4
3	0.0	30	1500	1.8	16	7.5	10	2000	1.2
4	0.0	40	2000	2.4	17	7.5	20	2500	1.8
5	0.0	50	2500	3.0	18	7.5	30	500	2.4
6	2.5	10	1000	1.8	19	7.5	40	1000	3.0
7	2.5	20	1500	2.4	20	7.5	50	1500	0.6
8	2.5	30	2000	3.0	21	10	10	2500	2.4
9	2.5	40	2500	0.6	22	10	20	500	3.0
10	2.5	50	500	1.2	23	10	30	1000	0.6
11	5.0	10	1500	3.0	24	10	40	1500	1.2
12	5.0	20	2000	0.6	25	10	50	2000	1.8
13	5.0	30	2500	1.2					

**Table 3:** TGA and DTG results of wood waste-filled PLA biocomposites.

Biocomposite	Temperature at Different Weight Loss (°C)				T_peak_ (°C)
	T_5_	T_25_	T_50_	T_75_	
PLA	327	348	357	365	360
PLA+2.5 wt.% wood waste	320	346	356	365	359
PLA+5 wt.% wood waste	312	337	350	359	354
PLA+7.5 wt.% wood waste	304	327	340	351	345
PLA+10 wt.% wood waste	300	325	338	348	342

**Table 4:** Volumetric wear and corresponding SN ratio.

Test Run	Volumetric Wear (cm^3^)	SN Ratio	Test Run	Volumetric Wear (cm^3^)	SN Ratio
1	0.0094	40.5374	14	0.0663	23.5697
2	0.0365	28.7541	15	0.0960	20.3546
3	0.0508	25.8827	16	0.0592	24.5536
4	0.0686	23.2735	17	0.0780	22.1581
5	0.1332	17.5099	18	0.1169	18.6437
6	0.0091	40.8192	19	0.1189	18.4964
7	0.0218	33.2309	20	0.1033	19.7180
8	0.0606	24.3505	21	0.0830	21.6184
9	0.0368	28.6830	22	0.0785	22.1026
10	0.0353	29.0445	23	0.0916	20.7621
11	0.0515	25.7639	24	0.1471	16.6477
12	0.0258	31.7676	25	0.1727	15.2542
13	0.0600	24.4370			

**Table 5:** SN ratio response table.

Level	A	B	C	D
I	27.19	30.66	26.78	28.29
II	31.23	27.60	25.84	24.69
III	25.18	22.82	24.25	25.54
IV	20.71	22.13	23.84	23.42
V	19.28	20.38	22.88	21.64
Delta	11.95	10.28	3.90	6.65
Rank	1	2	4	3

**Table 6:** Contribution ratio results.

Control Parameter	ℜ	${ƛ}_{i}$	ƛ	ψ
Wood waste	24.72	94.3663	201.5715	46.82
Normal load		72.7317		36.08
Sliding distance		9.8789		4.90
Sliding velocity		24.5946		12.20

## Data Availability

The data are available from the first author (T.S.) upon reasonable request.
